# QTL for Maize Midparent Heterosis in the Heterotic Pattern American Dent × European Flint under Corn Borer Pressure

**DOI:** 10.3389/fpls.2017.00573

**Published:** 2017-04-19

**Authors:** Luis F. Samayoa, Rosa A. Malvar, Ana Butrón

**Affiliations:** ^1^Misión Biológica de Galicia, CSICPontevedra, Spain

**Keywords:** *Zea mays*, yield, heterosis, quantitative trait loci, QTL

## Abstract

Despite the importance of heterosis and the efforts to comprehend this phenomenon, its molecular bases are still unknown. In this study, we intended to detect Quantitative trait loci (QTL) for mid-parent heterosis under infestation with the Mediterranean corn borer (MCB, Sesamia nonagrioides Lef.) using a North Carolina design III approach with a RIL population derived from a European flint inbred (EP42) × American dent inbred (A637) cross. QTL for heterosis of kernel yield have been positioned in regions corresponding to previously identified QTL for the same trait in different backgrounds. These results reinforce the high congruency of genes controlling heterosis across populations, even when populations have been developed from different heterotic patterns. A high percentage of genetic variation for mid-parent heterosis (Z_2_) for kernel yield could not be explained. Furthermore, genomic regions involved in heterosis for yield and plant height were not found despite the high genetic correlation between Z_2_ transformations for kernel yield and plant height. The moderate power in detecting QTL for mid-parent heterosis suggests that many genes with low augmented dominance effects contribute to the genetic architecture of mid-parent heterosis; dominance and additive-additive epistatic effects could also contribute to heterosis. However, results from this and previous studies suggest that the region 8.03–8.05 deserves special attention in future works in order to fine map loci involved in mid-parent heterosis for yield.

## Introduction

Heterosis is defined as the greater biomass, speed of development or fertility in heterozygotes, polyploids or hybrids compared to their parents (Schnable and Springer, 2013). Despite the importance of this phenomenom, its molecular basis is still unknown. In maize, mid-parent heterosis for yield, defined as the superiority of the hybrid over the mean of the parental inbreds, is particularly important because maize hybrids are extensively grown around the world. Most studies on the basis of maize mid-parent heterosis have been specifically focused in a unique heterotic pattern, Stiff Stalk Synthetic × Lancaster Sure Crop (Stuber et al., 1992; Frascaroli et al., 2007; Franco Garcia et al., 2008; Springer et al., 2009; Schon et al., 2010; Barber et al., 2012; Ding et al., 2014; Paschold et al., 2014), and conclusions from those studies cannot be generalized to other hybrids. Therefore, identifying quantitative trait loci (QTL) involved in heterosis in different genetic backgrounds is recommended as it has been rarely done (Tang et al., 2010; Lariepe et al., 2012; Guo et al., 2014). The heterotic pattern American dent × European Flint is particularly interesting because is the preferred pattern for hybrid development in Northern and Central Europe and Japan (Messmer et al., 1992; Moreno-Gonzalez et al., 2000; Technow et al., 2014).

The North Carolina design III and the immortilized F_2_ population design have been used to study the QTL involved in maize heterosis (Stuber et al., 1992; Cockerham and Zeng, 1996; Frascaroli et al., 2007; Tang et al., 2010; Guo et al., 2014). To detect QTL for mid-parent heterosis with an immortalized F_2_ population, a population of random crosses among RILs along with the RILs *per se* should be tested. Therefore, although the North Carolina design III with RILs does not allow to separate the contribution of dominance and epistasis to heterosis (Melchinger et al., 2007); it has been more extensively used because it does not require to test the RILs *per se*, but the backcrosses of those RILs to both parents (Frascaroli et al., 2007; Schon et al., 2010; He et al., 2012; Lariepe et al., 2012). The original North Carolina III mating design was developed by Comstock and Robinson (1948) to estimate the average level of dominance. This mating design has primarily been used in F_2_ populations to determine the effect of linkages on the estimates of additive and dominance variances, and the average level of dominance using progenies developed by backcrossing individual S_0_ plants of the F_2_ population to both parents (Hallauer and Miranda, 1981). In the present study, we attempted to detect QTL for mid-parent heterosis using a North Carolina design III approach with a RIL population derived from a cross between a European flint (EP42) and an American dent (A637) inbreds. The evaluations were conducted under infestation with the Mediterranean corn borer (MCB, *Sesamia nonagrioides* Lef.) to induce stalk damage, a major limiting factor for maize yield in the Mediterranean region. The cross EP42 × A637 presented high specific combining ability for yield under MCB infestation (Butron et al., 1999) and the backcrosses of RILs derived from this cross are ideal materials to study quantitative genetics of mid-parent heterosis for yield under infestation with MCB. Genes involved in heterosis under high pest pressure could be clearly different from those implicated in heterosis at optimal conditions because experimental data suggests that a suppression of defense response gene activities are important for generating the hybrid vigor phenotype due to the possible competition for resource distribution between plant defense and plant growth (Groszmann et al., 2015).

## Materials and methods

### Plant material and genotypic data

A population of 146 RILs derived from the cross of the European flint inbred line EP42 and the American dent inbred line A637 was developed (Samayoa et al., 2014) and 136 RILs were successfully backcrossed to both parents for mapping QTL for heterosis under infestation with MCB eggs. The mechanisms of resistance can be classified into three groups: antixenosis, antibiosis and tolerance (Painter, 1951). Inbreds EP42 and A637 were both classified as susceptible under MCB attack attending to their antibiosis levels, but A637 was tolerant (Butron et al., 1998) and showed good general combining ability for yield (Butron et al., 1999); while the cross EP42 × A637 presented significant and favorable specific combining abilities for yield and yield loss under MCB infestation (Butron et al., 1999). The linkage map used has been previously published by Samayoa et al. (2014).

### Phenotypic data

The backcrosses of the 136 RILs to both parents were evaluated at Pontevedra (42°24′ N, 8°38′ W, and 20 m above sea level), Spain, in 2010 and 2011. Backcrosses of RILs were arranged in 17 sets comprised each one of 8 RILs backcrossed to both parents, backcrosses of the same RIL were kept in adjacent plots. Sets and RILs within sets were randomized in each replication (two per trial). The trials were hand planted and each experimental plot consisted of one row spaced 0.8 m apart from the other row with 13 two-kernel hills spaced 0.18 m apart. Plots were overplanted and thinned, obtaining a final density of ~70,000 plant ha^−1^. The evaluations were performed under artificial infestation with eggs of MCB obtained at the Misión Biologica de Galicia by rearing MCB as described by Eizaguirre and Albajes (1992) and Khan and Saxena (1997). Five plants of each plot were infested with ~40 MCB eggs placed between the stem and the sheath of a basal leaf. Data collected were: days to silking computed as the number of days from planting to 50% of plants in the plot showing silks; ear and plant heights on five representative plants as the length (in cm) from the ground to the main ear and from the ground to the top of the plant, respectively; stem lodging defined as the percentage of plants in the plot with the stem broken below the main ear; kernel resistance to MCB attack on the ears of the infested plants collected at harvest according to a subjective visual resistance scale from 1 to 9 (1 indicates completely damaged and 9 indicates no damage); tunnel length as the average stem tunnel length made by borers on the five infested plants; kernel yield of the plot expressed in Mg ha^−1^ at 140 g H_2_O kg^−1^ (infested and non-infested plants were considered); and kernel moisture at harvest as g of water in 100 g of kernels.

### Statistical analysis

The individual analysis of each trial was made with the SAS mixed model procedure (PROC MIXED) (SAS Institute Inc, 2011) considering replications as random effects and sets and RILs within sets as fixed effects. The combined analysis across years was made considering years and replications as random effects. A Best linear unbiased estimate (BLUE) was obtained to estimate mean phenotypic values of each RIL backcrossed to P_1_ (EP42) and P_2_ (A637) in each year and across years.

To express the mid-parent heterosis as the sum of individual QTL effects, Melchinger et al. (2007) defined a new type of heterotic genetic effect ${\text{d}}_{i}^{*}$ that includes the dominance effect of QTL_i_ minus half the sum of its additive × additive epistatic interactions and was named augmented dominance effect. The same authors defined another genetic effect ${\text{a}}_{i}^{*}$ (named augmented additive effect) that includes the additive effect for QTL_i_ minus half the sum of dominance × additive epistatic interactions with all other QTL and corresponds exactly to the net contribution of QTL_i_ to parental differences. They also demonstrate that contrasts Z_1_ and Z_2_ in a modification of design III of Comstock and Robinson (Comstock and Robinson, 1952) using RILs instead of a F_2_ account for augmented additive and dominance effects, respectively. The linear transformations Z_1_ and Z_2_ are defined as follow:

Z_1_ = (H_1_ + H_2_)/2 (trait mean across each pair of RIL backcrosses),

Z_2_ = (H_1_ – H_2_)/2 (half the trait difference between each pair of RIL backcrosses),

H_1_ and H_2_ being the phenotypic observations on backcrosses of each RIL with the parental inbreds P_1_ and P_2_, respectively.

As a genome scan with Z_2_ localizes genomic regions affecting mid-parent heterosis and with Z_1_ identifies QTL that contribute to parental differences, QTL analyses were performed with values of Z_1_ and Z_2_ for each RIL and trait across years. In addition, a joint QTL analysis of Z_1_ and Z_2_ was implemented. Genetic variances and heritabilities (ĥ^2^) across environments were estimated for Z_1_ and Z_2_ for each trait on a family-mean basis as described previously by Holland et al. (2003). The augmented degree of dominance (D^*^) was estimated as the square root of the division between genetic variances for Z_2_ and Z_1_. The genetic correlation coefficients among Z_1_ tranformations for the different trais, among Z_2_ tranformations and between Z_1_ and Z_2_ for each specific trait were computed following Holland (2006).

The QTL analyses were performed with the software package PlabMQTL (Utz, 2012). Composite interval mapping approach (CIM) was conducted for the QTL detection and to estimate QTL effects. According to a previously executed permutation test with 1000 random reshuffles, a LOD threshold of 2.5 was chosen to declare significant a putative QTL assuming an experiment-wise error <30%. The phenotypic variance explained by the QTL model was estimated by the adjusted coefficient of determination ($R_{adj}^{2}$) which accounts for the total proportion of the phenotypic variance explained by all detected QTL in the final fit. Then, the phenotypic variance explained by an individual putative QTL_i_ was calculated as:

(1) $$\begin{array}{r} R_{i}^{2}=\;\frac{partR_{i}^{2}\;\times \;R_{adj}^{2}}{\sum_{i=1}^{n}partR_{i}^{2}} \end{array}$$

Where *n* = total of QTL in the final fit and $part\;R_{i}^{2}$ = partial coefficient of determination, estimated for the *i*th QTL detected (Zhu et al., 2004). The proportion of the genotypic variance ($\hat{p}$) explained by all detected QTL in final fit was estimated from the ratio $\hat{p}$ = $R_{adj}^{2}$/ĥ^2^ and the proportion of the genetic variance explained by each individual QTL*i* was estimated as ${\hat{p}}_{i}$ = $R_{i}^{2}$/ĥ^2^.

A 5-fold cross validation (CV) approach was employed for evaluating QTL mapping results (Utz et al., 2000). The data set (DS) was split at random into 5 data subsets; four subsets were combined to form de estimation set (ES) and the remaining subset formed the test set (TS). 1000 runs of CV were performed in order to determine the QTL frequency and bias of QTL effect estimate at the position of a QTL detected in the original data set (Melchinger et al., 2004). The proportion of the bias in the effect estimation of each individual QTL due to genotypic samplings was calculated as the difference between the average estimates in ES and TS divided by the estimate in ES.

## Results and discussion

Genetic variances for Z_1_ were significantly different from zero for all traits except for stalk lodging, tunnel length and kernel resistance; while genetic variances for Z_2_ were significantly different from zero for yield, yield-related traits, such as plant and ear heights, days to silking and stalk lodging (Table 1). The augmented degree of dominance was significantly higher than 1 for kernel yield, and significantly lower than 1 for days to silking, kernel moisture, and plant an ear heights. These results emphasize the great importance of heterosis for kernel yield and suggest that overdominance could play an important role as it has been reported in similar studies, although it should be taken into account that the augmented degree of dominance is biased by QTL linkage and epistasis and, consequently, does not exactly reflect gene action at individual loci (Melchinger et al., 2007; Schon et al., 2010; Lariepe et al., 2012). Heterosis was also important for yield-related traits, such as plant and ear heights and days to silking, but, as the augmented degree of dominance was significantly <1, dominance would be more important than over-dominance effects for these traits. In accordance to Lariepe et al. (2012), the higher importance of heterosis for days to silking in this cross compared to that in crosses between Reid and Lancaster inbreds could be due to the earliness of materials used and could emphasize the importance of studying heterosis in different backgrounds to get a better picture of the genetic architecture of the trait.

**Table 1:** **Genetic variance ± standard error and heritability estimations for the transformations Z_1_ and Z^a^_2_**.

	**Genetic variance**		**Genetic correlation**		**Heritability**	
**Trait**	**Z_1_**	**Z_2_**	**Z_1_–Z_2_**	D^*^	**Z_1_**	**Z_2_**
Stalk lodging	8.9 ± 5.43	0.41 ± 0.07¶	−0.01 ± 0.43	0.21	0.25	0.27¶
Days to silking	2.15 ± 0.39¶	0.53 ± 0.11¶	−0.27 ± 0.13¶	0.50#	0.71¶	0.63¶
Kernel moisture	0.39 ± 0.10¶	0.02 ± 0.04	0.19 ± 0.46	0.23#	0.54¶	0.10
Kernel yield	0.40 ± 0.12¶	0.86 ± 0.23¶	0.50 ± 0.15¶	1.47#	0.40¶	0.71¶
Plant height	89.05 ± 18.32¶	29.08 ± 5.16¶	−0.04 ± 0.13	0.57#	0.63¶	0.72¶
Ear height	35.08 ± 7.71¶	11.93 ± 2.22¶	−0.19 ± 0.14	0.58#	0.60¶	0.69¶
Tunnel length	5.76 ± 9.43	0.00	−	0.00	0.10	0.00
Kernel resistance	0.04 ± 0.02	0.00	−	0.00	0.24	0.00

a *Definitions of transformations Z_1_ and Z_2_ can be found in the materials and methods section*.

D^*^ is the augmented degree of dominance.

¶ *Significantly different from zero*.

# *Significantly different from 1 because the genotypic covariance between backcrosses to parent 1 and backcrosses to parent 2 significantly different from zero*.

Heritabilities for Z_1_ of agronomical traits, such as days to silking, kernel moisture, plant and ear heights and kernel yield were low to moderate; while heritabilities for Z_2_ were higher for plant and ear heights and kernel yield, slightly lower for days to silking, and considerably lower for kernel moisture than heritability estimates for the corresponding Z_1_ transformations (Table 1). Heritabilities did not differ from zero for Z_1_ and Z_2_ for related-resistance traits, although in a previous work heritability for tunnel length of the RILs *per se* was low but significantly different from zero (Samayoa et al., 2014). The discrepancy between the heritabilities computed for tunnel length of the RILs *per se* and for Z_1_ in this study could be consequence of opposite effects for additive × dominance epistasis and additivity. It could also be due to a higher experimental error in the current experiment based on the number of genotypes tested (backcrosses of RILs to both parents) that doubled the number of RILs *per se*. As additive × additive epistasis effects were not significant for tunnel length in a previous study (Butron et al., 2009), the absence of heritability for Z_2_ for tunnel length would indicate that dominance effects are not important for resistance to stem tunneling by MCB as it has been already suggested (Butron et al., 1999; Cartea et al., 1999).

As the environment × genotype interaction was not significant for Z_1_ and Z_2_ for yield and resistance-related traits, QTL analysis was not independently done for each environment (Data not shown). No QTL for Z_1_ for kernel yield were found, while two were found for Z_1_ for days to silking that explained the 32% of the genetic variation, and one for Z_1_ for kernel moisture, tunnel length, plant and ear heights and kernel resistance that explained the 17, 72, 20, 21, and 18% of genetic variation, respectively (Table 2). The QTL identified for Z_1_ for tunnel length at 9.03–9.04 co-localized with QTL for Z_1_ for both plant and ear heights. There was not a complete agreement between QTL detected for Z_1_ and QTL detected for the *per se* traits in a previous study (Samayoa et al., 2014); although evaluations of the RILs *per se* and backcrossed to the parental inbreds were performed in adjacent trials. Higher experimental errors expected in the current experiment compared to those in which the RILs *per se* were evaluated could be responsible for detecting fewer QTL for Z_1_. In addition, additive and additive × additive epistasis effects were confounded in the study by Samayoa et al. (2014); while additive and additive × dominance epistasis effects are undistinguished in the current study and both contribute to the augmented additive (a^*^) effect. Therefore, epistasis effects in this particular cross could be important enough to counteract additive effects. Previous studies have (Yan et al., 2006; Guo et al., 2014) already pointed out that digenic interactions at the two-locus level might play an important role in the genetic basis of maize heterosis. In any case, QTL for Z_1_ for tunnel length, and plant and ear heights positioned at 9.03–9.04, QTL for Z_1_ for kernel resistance positioned at 5.05–5.06, and QTL for Z_1_ for days to silking positioned at 8.05–8.06 were placed in the same bin or in adjacent bins to those where QTL for these traits *per se* were previously found (Samayoa et al., 2014) corroborating the presence of genes with additive effects for these traits in those regions.

**Table 2:** **Summary of QTL for transformations Z_1_ and Z^a^_2_ of several traits evaluated under infestation with MCB in the backcrosses of a RIL population derived from EP42 xA637**.

							**Effect^b^**						
**Parameter**	**Trait**	**Bin**	**cM**	**Interval**	**Left flanking marker**	**LOD**	**DS**	**ES**	**TS**	**Bias**	**$R_{i}^{2}$**	***p*****_*i*_**	**Frequency**
Z_2_	Stem lodging	6.02–6.03	41	28–51	umc1006	3.85	−2.19	−2.16	−1.62	0.25	11.4	41.9	0.47
	Kernel moisture	5.03	68	63–74	bnlg1046	6.24	0.19	0.19	0.18	0.08	10.4	100.0	0.95
	Kernel yield	1.02–1.03	71	54–90	bnlg1429	3.42	0.42	0.39	0.17	0.56	8.0	8.5	0.35
		3.04–3.05	93	83–114	phi029	3.21	0.31	0.34	0.14	0.58	5.9	6.2	0.20
		6.06	125	117–132	umc1424	3.20	0.32	0.36	0.25	0.30	8.4	8.9	0.57
		8.05	90	86–92	umc1309a	6.21	0.43	0.39	0.36	0.08	14.5	15.3	0.94
	Plant height	2.04–2.05	118	105–131	phi083	5.15	2.55	2.54	1.82	0.28	7.9	11.0	0.69
	Ear height	3.00–3.02	18	0–29	umc2101	6.32	−2.11	−2.18	−1.34	0.38	7.7	11.2	0.68
		5.03–5.04	85	74–94	umc1692	4.92	1.39	1.73	1.22	0.30	6.1	8.8	0.55
		5.05–5.06	140	125–161	umc1822	3.84	1.33	1.51	0.90	0.40	5.4	7.8	0.47
		8.01–8.02	23	0–42	umc1327	3.06	−1.97	−2.47	−0.74	0.70	5.1	7.4	0.16
		8.05–8.06	96	90–109	bnlg1812	3.26	1.60	1.62	0.81	0.50	9.0	13.0	0.53
Z_1_	Days to silking	7.03	25	18–29	mmc0411	2.93	0.67	0.77	0.31	0.60	7.0	9.8	0.36
		8.05–8.06	102	93–109	bnlg1812	7.56	1.37	1.36	1.24	0.08	15.5	21.7	0.86
	Kernel moisture	1.08–1.09	210	187–257	phi037	2.88	0.89	0.72	0.41	0.44	9.4	17.4	0.63
	Tunnel length	9.03–9.04	66	57–70	umc1267	3.41	−3.83	−4.05	−2.31	0.43	7.3	72.0	0.64
	Plant height	9.03	54	49–61	phi065	2.78	−6.54	−7.01	−6.33	0.10	12.5	19.7	0.77
	Ear height	9.03	54	50–59	phi065	7.93	−4.08	−4.51	−4.10	0.09	12.5	20.8	0.99
	Kernel resistance	5.05–5.06	111	110–118	umc1822	4.34	0.14	0.17	0.07	0.59	4.2	17.5	0.40

a *Definitions of transformations Z_1_ and Z_2_ can be found in the materials and methods' section*.

b *Effects estimated with the complete data set (DS), the estimation set (ES), the validation set (TS), and the proportion of the bias in the effect estimation of each individual QTL due to genotypic samplings calculated as the difference between the average estimates in ES and TS divided by the estimate in ES; ${\text{R}}_{i}^{2}$, the adjusted proportion of phenotypic variance explained by QTL_i_; p_i_, the adjusted proportion of the genetic variance explained by QTL_i_; Freq, percentage of cross-validation runs in which the QTL has been detected*.

Quantitative trait loci (QTL) for mid-parent heterosis (Z_2_ transformation) were found for the agronomical traits stem lodging, kernel moisture, kernel yield, plant height, and ear height and explained the 42, 100, 39, 11, and 48% of genetic variance, respectively (Table 2). QTLs for mid-parent heterosis for kernel yield were located at bins 1.02–1.03, 304–3.05, 6.06, and 8.05, were stable across years, and explained the 8.5, 6.2, 8.9, and 15.3 of the genetic variance, respectively (Tables 2, 3). The accuracy of effect estimation was low for QTL at 1.02–1.03 and 3.04–3.05, moderate for the QTL at 6.06 and high for the QTL at 8.05 because the estimations of bias proportions derived from cross-validation runs were 0.56, 0.58, 0.30, and 0.08, respectively. In addition, the level of occurrence of these QTLs in cross-validations was moderate and high for QTL at 6.06 and 8.05, respectively. When the Akaike information criterion (AIC), a less conservative criterion, is used instead of the modified Bayesian information criterion (BIC) for fitting a model that includes all QTLs for heterosis for kernel yield, the percentage of genetic variance explained increases to 52% and additional QTL were found at 2.04–2.06, 3.03, and 5.03–5.04 (Data not shown). Schon et al. (2010) also detected QTLs for the Z_2_ transformation for kernel yield in all these regions (same or adjacent bins) in at least one of three populations derived from Reid × Lancaster inbred crosses. However, Lariepe et al. (2012) detected nine QTL for Z_2_ for kernel yield that explained the 53% of phenotypic variance in crosses among an European flint and two dent inbreds, but only three of the genomic regions detected (the centromeric regions of chromosomes 3 and 5 and the long arm of chromosome 8) bear QTL for Z_2_ in the current study. Lastly, Tang et al. (2010) located QTL for mid-parent heterosis for yield in bins 1.01, 1.10, and 8.03 using an immortalized F_2_ population derived from the cross between a Chinese dent inbred and an exotic flint inbred (Tang et al., 2010; Guo et al., 2014). Then, QTL for kernel yield heterosis under high insect pressure were located in genomic regions known for including loci involved in heterosis at optimal conditions, but these results cannot serve to declare that, in general, competition between hybrid vigor and defense mechanisms does not exist because, in the current study, genetic variability for resistance-related traits was low. The number of QTLs detected for yield heterosis were lower than those detected by Schon et al. (2010) in dent crosses, similar to those detected by Lariepe et al. (2012) in early populations derived from European flint × dent and dent × dent crosses and larger than those detected by Tang et al. (2010) in a dent × flint cross. Assuming similar population sizes, the higher power to detect QTL for mid-parent heterosis for yield in the Reid × Lancaster heterotic pattern than in other heterotic patterns evaluated would relate to the larger magnitude of heterosis (absolute value) in the Reid × Lancaster pattern compared to the others.

**Table 3:** **Stability of QTLs for Z_1_ and Z_2_ transformation^a^ across years: effects and percentage of phenotypic variability explained by each QTL in each year**.

					**Effect (cm) ± standard error**		**Phenotypic variability**	
**Parameter**	**Trait**	**Bin**	**cM**	**Interval**	**2010**	**2011**	**2010**	**2011**
Z_2_	Stem lodging	6.02–6.03	41	28–51	−3.29 ± 0.84^*^	−0.84 ± 0.35^*^	8.8	2.8
	Kernel moisture	5.03	68	63–74	−0.28 ± 0.07^*^	−0.08 ± 0.06	11.0	0.0
	Kernel yield	1.02–1.03	71	54–90	0.40 ± 0.16^*^	0.43 ± 0.12^*^	2.6	5.3
		3.04–3.05	93	83–114	0.28 ± 0.13^*^	0.34 ± 0.11^*^	1.8	4.4
		6.06	125	117–132	0.34 ± 0.12^*^	0.30 ± 0.09^*^	3.4	4.5
		8.05	90	86–92	0.44 ± 0.11^*^	0.44 ± 0.09^*^	5.8	9.4
	Plant height	2.04–2.05	118	105–131	2.48 ± 0.73^*^	2.52 ± 0.87^*^	6.5	4.6
	Ear height	3.00–3.02	18	0–29	−2.11 ± 0.48^*^	−2.13 ± 0.50^*^	7.8	7.7
		5.03–5.04	85	74–94	1.39 ± 0.37^*^	1.40 ± 0.38^*^	6.1	5.9
		5.05–5.06	140	125–161	1.33 ± 0.37^*^	1.37 ± 0.38^*^	5.4	5.5
		8.01–8.02	23	0–42	−1.97 ± 0.57^*^	−1.94 ± 0.57^*^	5.1	4.8
		8.05–8.06	96	90–109	1.60 ± 0.34^*^	1.66 ± 0.35^*^	9.0	9.2
Z_1_	Days to silking	7.03	25	18–29	0.54 ± 0.19^*^	0.77 ± 0.25^*^	4.5	5.6
		8.05–8.06	102	93–109	1.13 ± 0.25^*^	1.66 ± 0.32^*^	10.7	13.8
	Kernel moisture	1.08–1.09	210	187–257	1.43 ± 0.33^*^	0.35 ± 0.20	10.8	0.0
	Tunnel length	9.03–9.04	66	57–70	−4.23 ± 1.38^*^	−3.69 ± 1.52^*^	5.1	2.9
	Plant height	9.03	54	49–61	−4.72 ± 1.69^*^	−8.47 ± 1.43^*^	4.1	20.1
	Ear height	9.03	54	50–59	−4.08 ± 0.88^*^	−4.33 ± 0.89^*^	12.5	14.0
	Kernel resistance	5.05–5.06	111	110–118	0.09 ± 0.08	0.15 ± 0.06^*^	0.0	2.8

a *Definitions of transformations Z_1_ and Z_2_ can be found in the materials and methods' section*.

* *Significantly different from zero at 0.05 probability level*.

Results from this and previous studies suggest that the region 8.03–8.05 deserves special attention in future works in order to fine map loci involved in mid-parent heterosis for yield. This genomic region harbors QTL with important additive augmented (a^*^) effects for days to silking and dominance augmented (d^*^) effects for ear height and kernel yield in this study. Previously, in the same region, significant QTL for Z_2_ for yield were identified in several populations (Schon et al., 2010; Tang et al., 2010; Lariepe et al., 2012) and is one of the genomic regions with high density of QTL peaks for yield based on a MetaQTL analysis involving results from 44 manuscripts (Martinez et al., 2016). In addition, Samayoa et al. (2014) also detected important QTL for days to silking and plant and ear heights in the same region. This region presents high homology with the region of chromosome 6 [these regions correspond to duplicated regions of an ancient tetraploid (Schnable et al., 2011)] where a QTL for yield heterosis has been located in the current study and in that performed by Schon et al. (2010).

No overlapping QTL for Z_1_ and Z_2_ for kernel yield were found although genetic correlation between both parameters was moderate (0.50) suggesting that genomic regions containing QTL with augmented additive and dominance effects for yield went undetected due to the low magnitude of genetic effects (Table 4). In a similar way, although the correlation coefficients between Z_1_ for yield and Z_1_ for ear height and between Z_2_ for yield and Z_2_ for plant and ear heights were high and significant (Table 4), only two QTL for Z_2_ for kernel yield (at 8.05 and 5.03–5.04) co-localized with QTL for Z_2_ for ear height. These facts along with the lack of power for detecting QTL for Z_1_ for kernel yield and Z_2_ for days to silking, although genetic variability for these transformations significantly differed from zero (Table 1), suggest that many genes with low augmented additive and/or dominance effects can contribute to kernel yield and mid-parent heterosis for yield, at least in this particular cross. In addition, the augmented dominance effects of some loci that contribute to heterosis could be consequence of additive-additive epistatic effects. Lariepe et al. (2012) showed that when a QTL for Z_2_ for yield overlap with a QTL for yield with no significant dominance effect, the QTL probably presents significant additive-by-additive interaction effects. Therefore, the QTL detected for Z_2_ for yield at 5.03–5.04 that was already reported as important for yield *per se* (Samayoa et al., 2014) could rather contribute to Z_2_ due to additive-by-additive interactions than to dominance effects. The significant contribution of additive-by-additive interactions to heterosis in this particular cross was already reported in a previous study (Butron et al., 2009).

**Table 4:** **Genetic correlation coefficients ± standard errors between Z_1_ and Z_2_ for yield and the corresponding Z_1_ and Z_2_ transformations^a^ for other traits**.

**Z_1_**	**Yield**	**Z_2_**	**Yield**
Stalk lodging	0.44 ± 0.35	Stalk lodging	−0.55 ± 0.22^*^
Days to silking	0.36 ± 0.20	Days to silking	−0.27 ± 0.12^*^
Kernel moisture	0.71 ± 0.23^*^	Kernel moisture	0.91 ± 0.86
Plant height	0.36 ± 0.18^*^	Plant height	0.88 ± 0.06^*^
Ear height	0.83 ± 0.17^*^	Ear height	0.75 ± 0.08^*^
Tunnel length	0.15 ± 0.55	Tunnel length	−
Kernel resistance	0.51 ± 0.32	Kernel resistance	−

a *Definitions of transformations Z_1_ and Z_2_ can be found in the materials and methods section*.

* *Significantly different from zero at 0.05 probability level*.

As conclusions, the low to moderate power of the experiment to detect QTL for mid-parent heterosis suggests that many genes with low augmented dominance effects can contribute to the genetic architecture of mid-parent heterosis. Furthermore, dominance and additive-additive epistatic effects could also contribute to heterosis. The genomic regions detected for mid-parent heterosis for kernel yield in this particular cross (European flint × American dent inbreds) had been detected as significant for heterosis in other heterotic patterns reinforcing the idea of high congruency of heterosis genes across populations. Results from this and previous studies suggest that the region 8.03–8.05 deserves special attention in future works in order to fine map loci involved in mid-parent heterosis for yield.

## Author contributions

RM and AB designed the work; LS did the collection and statistical analysis of data; and AB drafted the manuscript that was critically revised by LS and RM.

## Funding

This work was supported by the National Plan for Research and Development of Spain (projects AGL2012-33415 and AGL2015-67313-C2-1-R, both funded in part by the European Regional Development Fund).

### Conflict of interest statement

The authors declare that the research was conducted in the absence of any commercial or financial relationships that could be construed as a potential conflict of interest.

## Abbreviations

MCB: Mediterranean corn borer
QTL: Quantitative trait loci
RIL: Recombinant inbred line
CV: Cross validation
DS: Data set
ES: Estimation set
TS: Test set.
